# Mitigating Illumination-, Leaf-, and View-Angle Dependencies in Hyperspectral Imaging Using Polarimetry

**DOI:** 10.34133/plantphenomics.0157

**Published:** 2024-03-22

**Authors:** Daniel Krafft, Clifton G. Scarboro, William Hsieh, Colleen Doherty, Peter Balint-Kurti, Michael Kudenov

**Affiliations:** ^1^ Department of Electrical and Computer Engineering, North Carolina State University, Raleigh, NC, USA.; ^2^ NC Plant Sciences Initiative, North Carolina State University, Raleigh, NC, USA.; ^3^ Department of Molecular and Structural Biochemistry, North Carolina State University, Raleigh, NC, USA.; ^4^ Department of Entomology and Plant Pathology, North Carolina State University, Box 7616, Raleigh, NC 27695, USA.; ^5^ Plant Science Research Unit, USDA-ARS, Raleigh, NC 27695, USA.

## Abstract

Automation of plant phenotyping using data from high-dimensional imaging sensors is on the forefront of agricultural research for its potential to improve seasonal yield by monitoring crop health and accelerating breeding programs. A common challenge when capturing images in the field relates to the spectral reflection of sunlight (glare) from crop leaves that, at certain solar incidences and sensor viewing angles, presents unwanted signals. The research presented here involves the convergence of 2 parallel projects to develop a facile algorithm that can use polarization data to decouple light reflected from the surface of the leaves and light scattered from the leaf’s tissue.

The first project is a mast-mounted hyperspectral imaging polarimeter (HIP) that can image a maize field across multiple diurnal cycles throughout a growing season. The second project is a multistatic fiber-based Mueller matrix bidirectional reflectance distribution function (mmBRDF) instrument which measures the polarized light-scattering behavior of individual maize leaves. The mmBRDF data was fitted to an existing model, which outputs parameters that were used to run simulations. The simulated data were then used to train a shallow neural network which works by comparing unpolarized 2-band vegetation index (VI) with linearly polarized data from the low-reflectivity bands of the VI. Using GNDVI and red-edge reflection ratio we saw an improvement of an order of magnitude or more in the mean error (*ϵ*) and a reduction spanning 1.5 to 2.7 in their standard deviation (*ϵ*_*σ*_) after applying the correction network on the HIP sensor data.

## Introduction

Plant phenotyping has the potential to automate agricultural processes and improve crop diagnostics for farmers. Rapid increases in global populations coupled with a decrease in cultivated land area, intensification of climate change, and water shortages pose a tremendous challenge to the agricultural sector [1,2]. Researchers worldwide are using phenomics to accelerate breeding programs, monitor crop health, and generate quality control standards from a small scale (e.g., biochemical and molecular) to a large scale (e.g., whole plant and ecosystem). With all this research, there is still a large gap between the quality of phenotyping achieved in a controlled environment (e.g., greenhouses and grow rooms) and phenotyping in the field.

A common challenge when measuring leaf or canopy color in the field relates to the bidirectional reflectance distribution function (BRDF) of the plants’ leaves being analyzed. Depending on solar incidence and viewing angles, spectral reflection of sunlight (i.e., glare and hot spots) from crop leaves presents unwanted signals [3]. This glare is relatively bright and generally masks the leaf’s true color, compromising the reliability of sampled data. Recent studies sought to characterize canopy-level impacts of BRDF in outdoor remote phenotyping [4–6] and existing methods of correcting glare as a confounding factor have included light scattering simulations [7,8], 3-dimensional sensor fusion [9], or taking several measurements in time series [10]; however, they either require complex computations, modeling, and expertise—or many, possibly impractical time-series measurements taken at different viewing and illumination geometries—to implement effectively. Light polarization has also emerged as a method to determine a surface’s orientation; however, the demonstrated techniques still require time-series measurements or fixed viewing- and object-normal vectors [11,12]. Beyond this, polarized light has been leveraged specifically for leaf phenotyping in the laboratory, under controlled illumination and sensing conditions [13–16], but a broadly accessible leaf-level BRDF correction strategy using polarization has not yet been demonstrated in uncontrolled field settings.

Converse to prior techniques, we aim to create a single-frame (single-measurement) glare color correction technique using polarized BRDF (pBRDF) leaf reflection models and polarization-sensitive spectral measurements. To simplify our approach and broaden the initial adoption of our proposed glare correction algorithm, we have chosen to down-sample our 200 wavelength band spectropolarimeter data to simpler 2-band vegetation indices (VIs)—green normalized difference vegetation index (GNDVI) and red-edge reflectance ratio (RERR). Using these metrics, we developed and validated an algorithm to better estimate leaf color in the presence of glare.

To establish an accessible correction technique, we leveraged 2 primary sensors: a multistatic fiber-based (MFB) BRDF polarimeter [17] to characterize maize leaves in the lab, and a hyperspectral imaging polarimeter (HIP) [18] for quantifying the diurnal variation of leaf and canopy reflectance in the field. The following sections will introduce some relevant background information and polarimetry before defining the polarization bidirectional reflectance distribution function (pBRDF) and its application to remove glare and improve leave color estimation in field trials. In Materials and Methods, we introduce our experimental design, sensor technologies, pBRDF maize leaf modeling, correction model development, simulations, and error analysis. The experimental results are presented in Results including pBRDF model outputs, correction model simulations, ground truth data, and correction model validation using field trial data. In Discussion, we discuss the results of our polarization BRDF-based correction network and conclude the study with a recap on work achieved, potential impacts, and a look into the future direction of this work.

### Background

While measurement of a sample’s spectral reflectance can be used to identify a material’s chemical composition, polarization information can be used to identify structural or geometric features (e.g., surface roughness, shape, and scattering angle) of the sample. Taking images in real-world environments, such as a maize field, one will observe varying amounts of glare. This glare results from the interaction of incident light with the air–surface boundary of the leaf and, while present at all illumination and view angles, is generally most prominent in the specular direction. Most of the light being reflected is not interacting with the structure of the leaf. Rather, the sensor is predominantly measuring the source’s spectrum (i.e., the sun). Our experiments are designed based on the fact that this glare is generally more polarized than regions with less observable glare. We hypothesize that this glare can be corrected by considering the pBRDF of maize leaves and the polarization state of the field sample image, along with the illumination and view angles from the field setup. In effect, the correction process relies on determining the surface normal of leaves within the canopy and correcting the glare by considering the Mueller matrix bidirectional reflectance distribution function (mmBRDF) of individual maize leaves.

### Polarimetry

Polarimetry is the measurement and interpretation of the polarization of transverse (electromagnetic) waves. Polarization tends to provide information that is largely uncorrelated with spectral and intensity images and thus has the potential to enhance many fields of optical metrology [19,20]. It is with this in mind that many researchers have been working to develop spectropolarimeters capable of measuring spectral and polarization information [16,21–31].

Stokes parameters are a set of values that describe the polarization state of electromagnetic radiation and are defined as

$$S\left(\lambda \right)={\left[S_{0}\;S_{1}\;S_{2}\;S_{3}\right]}^{T},$$ (1)

where *S*_0_ describes the total (unpolarized) intensity of the incident light, *S*_1_, is the intensity difference between vertical and horizontal linear polarization states, *S*_2_ is the intensity difference between +45 and −45 linear polarization states, *S*_3_ is the intensity difference between right and left circular polarization states, *T* is the transpose operation, and *λ* is the wavelength [32]. Polarization analyzing optics, such as rotating linear retarders and linear polarizers, can be employed to measure **S**.

When light is reflected from a surface, the light–matter interaction can be characterized by changes within the reflected Stokes vector. A Mueller matrix models this input-output relationship by

$$S_{o}\left(\lambda \right)=MS_{i}\left(\lambda \right),$$ (2)

where **S**_**i**_ is the incident Stokes vector, **M** expresses a 4 × 4 Mueller matrix of the sample, and **S**_**o**_ is the reflected, transmitted, or absorbed (output) Stokes vector.

Since our experiments only measure linearly polarized light, *S*_3_ is not considered. In this case, **M** can be reduced to a 3 × 3 matrix and **S** contains only *S*_0_, *S*_1_, and *S*_2_ [32,33]. From these parameters, the degree of linear polarization (DoLP) can be calculated by

$$\text{DoLP}=\sqrt{{\left(S_{1}\right)}^{2}+{\left(S_{2}\right)}^{2}}/S_{0}.$$ (3)

**Fig. 1.**
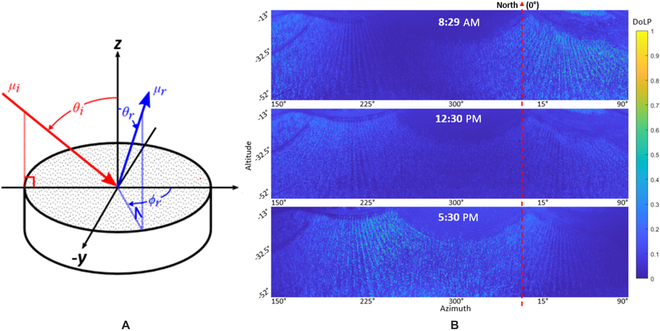
BRDF angular conventions and DoLP plots. (A) Angular conventions used for BRDF modeling. (B) Three example DoLP images of the field sampled at 8:29 AM, 12:30 PM, and 5:30 PM. Note how the bright sections with high DoLP shifts across the field as the position of the sun changes throughout the day.

Angular conventions used for backward-scattering mmBRDF models are presented in Fig. 1A. Incident light is represented by *μ_i_* with an altitude angle of incidence *θ_i_*. Reflected light is represented by *μ_r_* with an altitude and azimuth angle of reflection, *θ_r_* and *ϕ_r_*, respectively. In our mmBRDF models, incident light represents the sun’s position in the sky (time of day) and the angles of reflected light are representative of the camera (or observer) viewing angles. When we vary these values, the observed spectral and polarization states change. This is a property of mmBRDF which is illustrated in Fig. 1B where we see the DoLP change based on time of day and viewing angle of the sensor.

DoLP spans 0 (unpolarized) to 1 (fully polarized) and characterizes the proportion of energy, received by the sensor, that is linearly polarized. The DoLP changes continually throughout the day based on the leaf angle, incident sunlight angle, and view angle and is a key metric of our modeling efforts. Three example images of DoLP are displayed in Fig. 1B below for different times of the day. The amount of specular reflected light observed is related to the angle of incident light and the view angle of the sensor system. At lower angles of incidence, when the sun is lower in the sky, we observe a higher DoLP in the image. In the middle of the day, when the sun is highest in the sky, a lower DoLP is observed. It is assumed that regions containing glare are more polarized than others and can therefore be used as a metric that drives a bidirectional reflectance-based correction.

### Polarization BRDF

Polarization BRDF describes the polarized reflectance of a target as a function of illumination, polarization, and viewing geometries [17,34–36]. Reflected light can be modeled as a combination of specular (mirror-like) scattering and Lambertian (diffuse) scattering. In the context of plant phenotyping, specular reflection is unwanted light that mimics the incident light’s spectrum [11]. Conversely, Lambertian reflection consists of light that is interacting with the leaf’s structure and carries useful information about the plants’ status (e.g., pigment concentration(s), stress response, photosynthetic status, etc.). By considering the nature of a plant’s BRDF and the polarization state of the measured light, we can correct the Lambertian reflection in the presence of a specular component.

A property of a BRDF is that there is an intensity difference between forward- and backward-scattering light. By placing light and polarization detectors around a wide range of angles, a mmBRDF measurement allows us to observe those differences across a wide range of samples and treatments [17]. In the field, we know the illumination angle of the sun and the view angle of the camera. Using the mmBRDF model of a leaf, we can infer the angle of the leaf normal relative to the camera system. This information provides us with the potential to isolate Lambertian-reflected data from a sample image by removing the specularly-reflected glare.

## Materials and Methods

The research presented here is the convergence of 2 parallel projects. The first project involved field trials using our HIP sensor to gather data from a maize field across the season [18]. The second project sought to generate a light scattering model of individual maize leaves using our MFB mmBRDF instrument [17]. Data from mmBRDF measurements were then fitted to an existing light scattering model using SCATMECH [36]. Each mmBRDF model returned a set of parameters that were used in Monte Carlo simulations to generate a large dataset across a wide range of solar incidence-, leaf-, and viewing angles; which were chosen to mimic field trial conditions observed by the HIP sensor. This dataset was then used to train a shallow neural network (NN) which performs mmBRDF correction. Finally, data acquired from HIP field trials were curated and sent into the NN to perform leaf-level mmBRDF correction.

### Field trials and experimental design

**Fig. 2.**
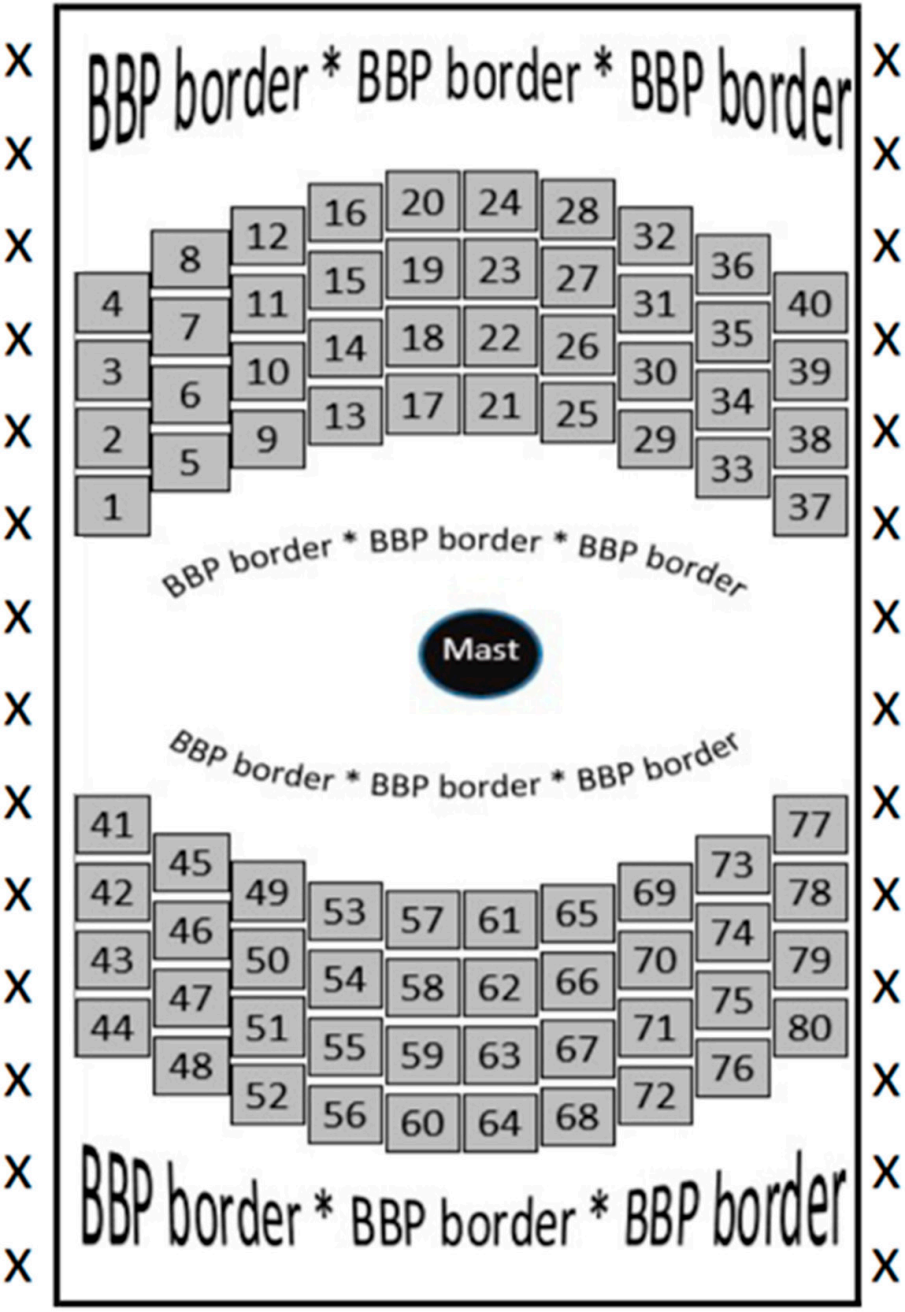
Plot map from 2021 field trials.

Field trials consisted of 2 replicates with 40 plots of the maize inbred line B73, which were planted at a North Carolina State University Central Crops Research Station in Clayton, NC (35.664026, −78.508504). Figure 2 presents the field map for our trials where BBP is a purple variety of maize that was used to establish the fields’ border rows and boundaries. The HIP camera system was mounted on a mast in the middle of the field and diurnal measurements were captured, from sunrise to sunset, across the field once per hour. Only data from growth stages R1 to R6 were considered for this experiment, in an effort to focus our study on mature, adult maize leaves. Since the incident and viewing geometries are well defined on clear and sunny days, we elected to focus on data gathered from sunny days. As opposed to cloudy days where the incident geometry is less defined [37].

**Fig. 3.**
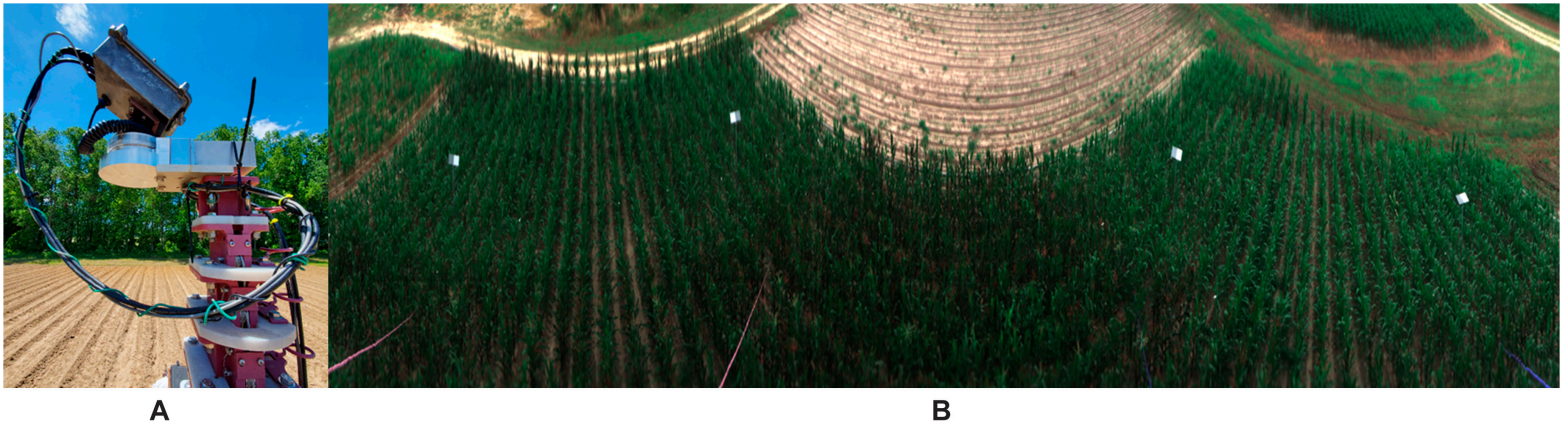
HIP sensor and sample FOV. (A) Image of the mast-mounted HIP camera system. (B) Composite RGB image demonstrating the camera’s FOV. The 4 gray squares in the FOV are (secondary standard) reference tiles used for spectral calibration.

The HIP uses a microgrid polarimetric camera to measure the linear polarization states of light [*S*_1_,*S*_2_], along with *S*_0_. Figure 3A and B below shows an image of the HIP camera system and a sample composite RGB image of the field of view (FOV), respectively. The HIP sensor design and implementation is discussed in more detail in Hyperspectral imaging polarimeter and HIP calibration below.

Finally, 4 secondary reflectance (or reference) tiles were placed in the field to provide the HIP sensor with a downwelling radiance measurement. The tiles were 600 × 600 cm^2^ and had one side painted dark gray, while the other side was painted light gray. The tiles were mounted on 3-m poles such that the growing maize does not block the camera’s line of sight. We see these 4 reference tiles in the composite RGB image in Fig. 3B. On a sunny day, early in the season, we carried a large 50% reflective Spectralon (sintered polytetrafluoroethylene; from LabSphere) calibration tile into the field for a single scan. Having a known spectral reflectance from the near-ultraviolet to the near-infrared and being relatively Lambertian, this primary standard was used to measure and reference our secondary standards [38]. Calibration of the 4 reference tiles using the Spectralon reference was calculated as *ρ_tile_*. For each sample captured throughout the season, the spectra were calculated by *ρ_sample_* = *ρ_tile_I_sample_*/*I_tile_*.

Each sample of the field consisted of 3 data cubes (*S*_0_, *S*_1_, and *S*_2_), each with spatial and spectral dimensions (x, y, *λ*). The hyperspectral band is sampled for wavelengths spanning 500 nm (blue) to 800 nm (near infrared) with a spectral resolution of (∼ 4 nm). Performance of the algorithm before and after correction is evaluated by considering a 2-band vegetation index (VI) metric such as GNDVI, and RERR [39]. In this paper, RERR is defined similarly to normalized difference vegetation index (NDVI) with an adjustment in the red band such that it was shifted to longer wavelengths (690 to 710 nm). This was done to avoid saturation of the ground truth data, where the typical NDVI red band (660 to 680 nm) transmission ratio was close to zero.

The experimental setup assumes that the maize was unstressed (or minimally stressed) and the VI is spatially and temporally uniform across the field between the R1 and R6 growth stages. Liebisch et. al. [40] performed aerial phenotyping of maize across the season using an RGB, NDVI, and high-resolution thermal camera. In their work, they also found the NDVI of maize to be fairly consistent across the season. Our uniformity assumption was further supported with ground truth data, captured using a handheld device that is discussed in the following section. Given the validity of the above assumptions, spatial and temporal differences in our VIs across the field can be attributed to bidirectional reflectance.

### Spectral ground truth data

Ground truth data was acquired using a commercial off-the-shelf fiber spectrometer that was operated in transmission-mode. The device consisted of a white light-emitting diode light source (Thorlabs 500 to 850 nm), a lens to focus light onto the sample, and an Ocean Optics spectrometer was positioned on the back surface of the leaf to capture the transmitted light. The spectrometer was controlled using a Raspberry Pi, and a Raspberry Pi camera was configured to read QR codes, containing plot indices, in the field and to automatically associate the measured spectra with these indices.

Several times throughout the season, this device was used to measure multiple leaves in each of the 80 field plots. These data were then used to calculate a 2-band metric (i.e., RERR and GNDVI) across the field for the given day to establish a target value for polarization correction and to evaluate the consistency of these metrics across the season. It was assumed for this research that after the R1 growth stage, the VI remains relatively stable through R6 (physiological maturity) [40]. Each measurement consisted of selecting the 4th leaf from the tassel from 5 plants contained within the center row of each plot.

### Hyperspectral imaging polarimeter

The HIP is a prism-based pushbroom imaging spectropolarimeter. The HIP design follows similarly to that described in Ref. [18] with a few adjustments. The polarizing optics (AQWP, R1, R2, LP) and Allied Vision Technologies FPA have been removed, and instead, a microgrid polarization camera (Lucid Vision Phoenix 5.0MP Polarization Model with a Sony IMX250MZR/MYR) was used. This monochromatic camera uses linear wire grid polarizers at 0°, 45°, 90°, and 135° orientations for each superpixel to measure polarized light, making the new system a division of focal plane polarimeter (DoFP) [41]. Sample light enters the system through a 3-mm-diameter aperture stop in front of an objective lens which focuses the light onto a 15-μm-wide, 10-mm-long slit before being dispersed by an N-SF11 and CaF2 equilateral prism pair. In this configuration, spectral information was measured across the camera’s *x*-axis, vertical spatial information was measured across the *y*-axis, and polarization information was measured at each *λ*, *y* coordinate with the pixelated microgrid camera’s superpixel [19,42,43]. To obtain pushbroom scanning and achieve a horizontal spatial axis, the camera rotated along the azimuth to scan the field, capturing a spatial column of pixels with each measurement. These columns were stitched together in postprocessing to generate 3 data cubes, each containing *x*, *y*, *λ* dimensions, for the linear Stokes parameters *S*_0_, *S*_1_, and *S*_2_.

### HIP calibration

There are 3 areas to be addressed when calibrating the HIP sensor: spectral, radiometric, and polarimetric. To perform the system’s spectral calibration, a monochromator was employed to vary the incident light spectrum from 425 to 800 nm in steps of 25 nm. Incident light from a tungsten halogen lamp was input into a monochromator, which dispersed and redirected the source light into an integrating sphere. This light was then imaged and recorded by the sensor. Additionally, a dark frame was acquired by blocking the entrance aperture for a single measurement. The dark frame was used to remove background noise from the monochromatic data. A detailed description of the spectral calibration process can be found in Ref. [18].

The sensor’s radiometric calibration was achieved via flat-field correction (FFC). FFC is the process of correcting the nonuniform pixel-to-pixel intensity of an image. FFC corrects the differences of light sensitivity between the pixel sensors of a camera detector array, differences of illumination intensities in the FOV, and differences in the transmission of light through the lens (vignetting). The base equation for FFC takes the form of

$$I_{C}=I_{R}*\rho +O,$$ (4)

where *I_C_* is the pixel’s corrected intensity, *I_R_* is the original (raw) intensity, *O* is a radiometric offset, and *ρ* is the responsivity. FFC is achieved by imaging a known source through an integrating sphere to generate a uniform signal such that each pixel should see the same intensity. Using this data, FFC adjusts each pixel of the raw images such that they all provide the same output *I_C_* given the same input [44,45].

Finally, a polarimetric calibration was conducted. The Stokes vector cannot be directly measured, therefore, to calculate the Stokes parameters, several individual measurements must be performed and then combined [32,33]. Polarimetric calibration of a DoFP can be achieved using a data reduction matrix. The goal of polarimetric calibration by data reduction matrix is characterizing the system’s measurement matrix **W**. In general, the detected power in the system can be represented by

P=WS, (5)

where **P** is the detected power and **S** is the Stokes vector incident on the sensor. Using linear operator theory, we can solve for **S** by

$$S=W^{-1}P.$$ (6)

where the inverse of **W** may be taken by pseudoinverse if it is not symmetric.

**Fig. 4.**
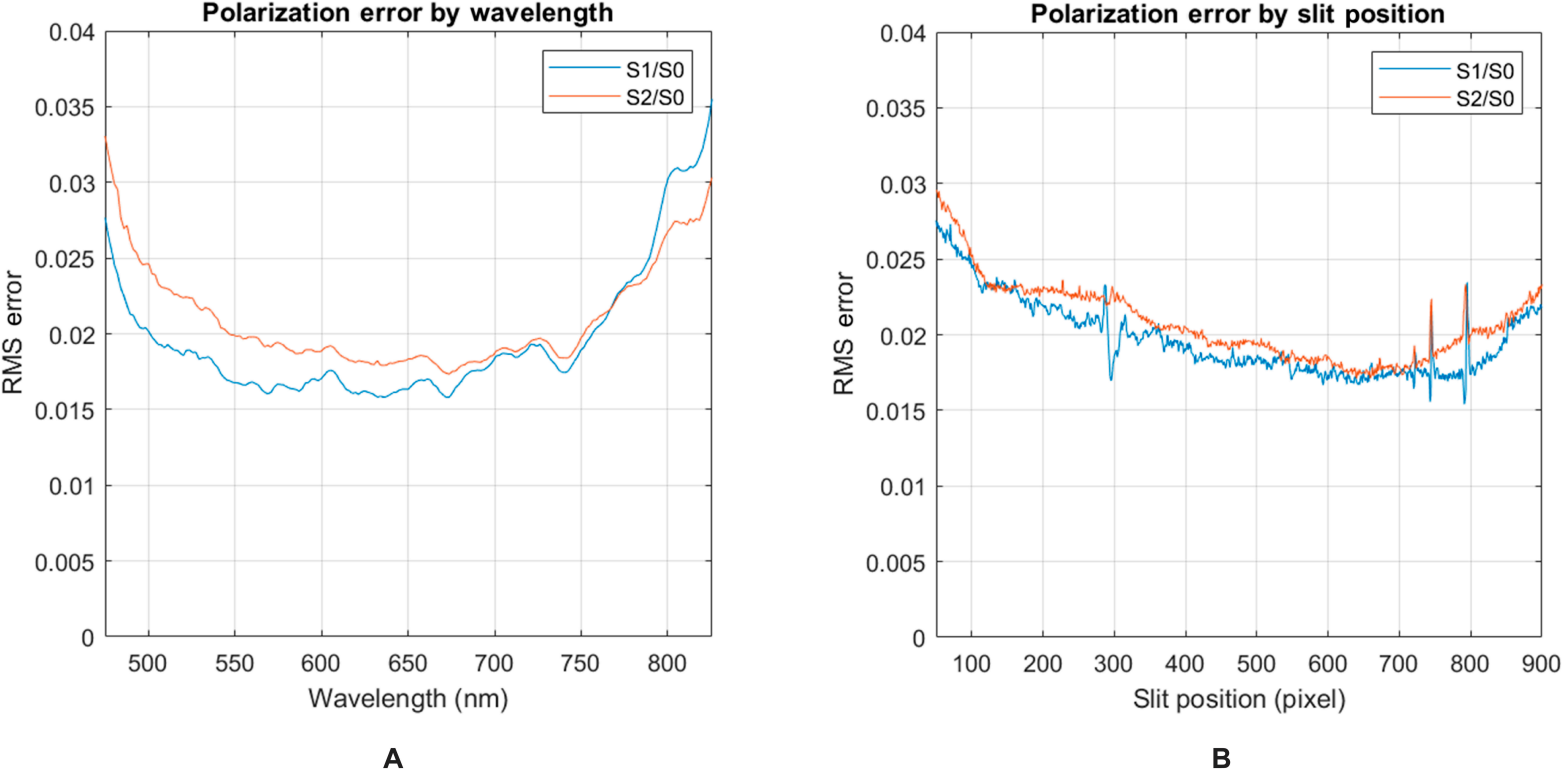
Polarization error plots after calibration. (A) Polarization error with respect to wavelength (across the x-axis of the detector array). (B) Polarization error with respect to slit position (across the y-axis of the detector array).

**Fig. 5.**
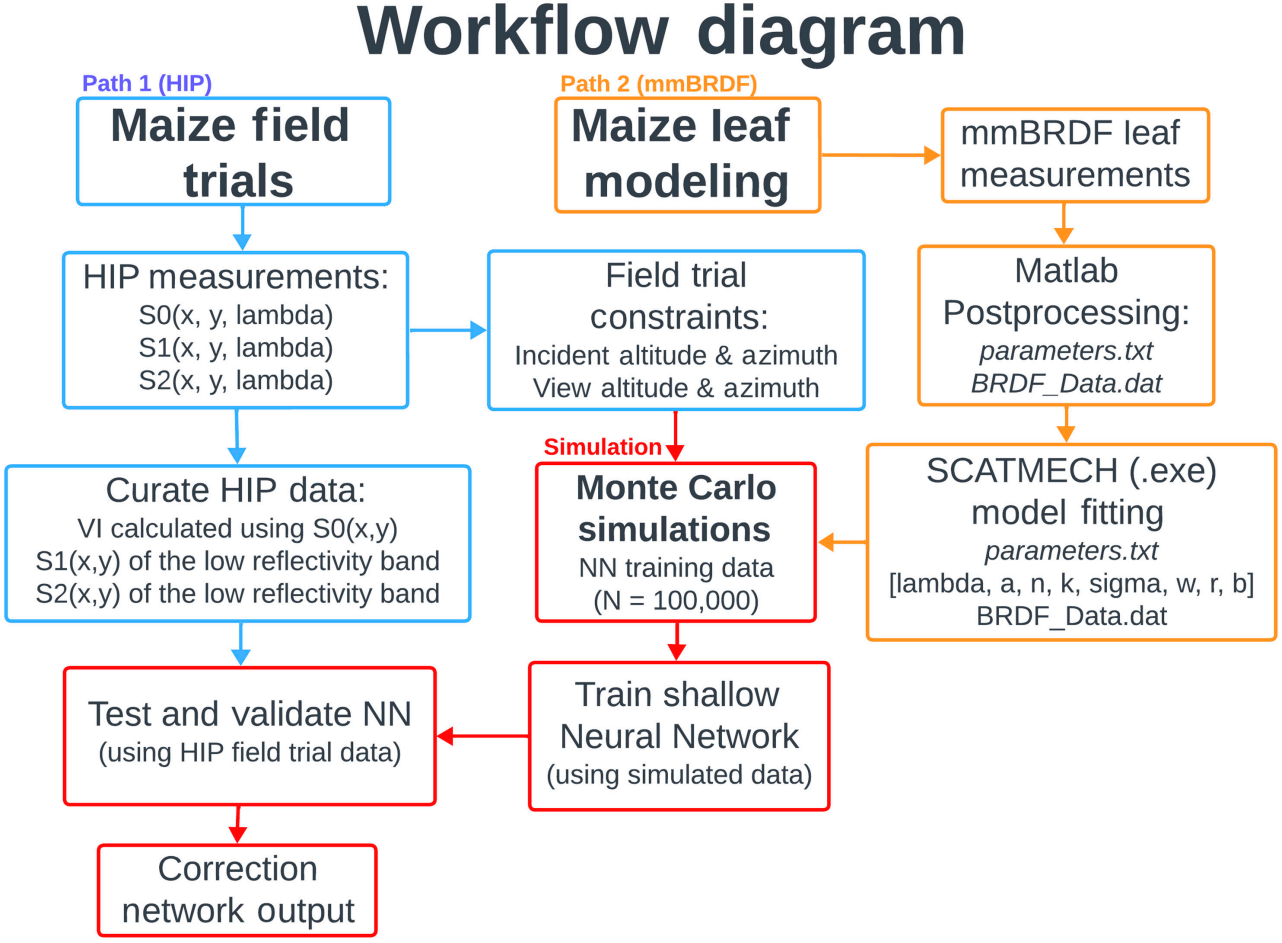
Workflow diagram presenting a high-level summary of the data paths and order of operations used to merge the field trial data with the mmBRDF-SCATMECH modeled data with Monet Carlo simulations. Here, the blue path represents field trial data from the HIP; the orange path represents mmBRDF measurements and model-fitting; and the red path shows simulation, NN training, and validation with real-world data acquired from our field trials.

As described in Ref. [45], the measurement matrix was obtained using a similar experimental configuration to spectral calibration, except instead of a monochromator, a spectrally broadband light source was used with a polarization state generator placed between the integrating sphere and the sensor. Measurements were taken with different known polarization states such that **S** is a known Stokes vector. We then solved for **W** in Eq. 5 above. Once acquired, the measurement matrix for each pixel on the camera was saved and used for all future polarization measurements captured in the field. To characterize the polarization error present in the system, both the *x*-axis (*λ*) and *y*−axis must be considered. This was achieved by measuring the root mean square error of the detected polarization state with respect to wavelength and slit position, per Figs. 4 and 5, respectively, and comparing to the polarization state generator theoretical polarization. A more detailed review of polarimetric calibration steps can be found in Tyo [19], Kudenov [45], and Goldstein [33].

### pBRDF instrument and SCATMECH model fitting

In addition to the field materials, a separate group of maize plants were grown in a greenhouse for more rigorous mmBRDF measurements and modeling. A total of 3 B73 maize plants were used for these experiments. Each plant was grown to R1 and the leaves were measured while still attached to the plant to minimize changes. Currently, we are focusing on B73, but in the future, we will introduce other varieties of maize, as well as other crops (soybean, wheat, etc.), in an effort to establish a library of mmBRDF models and parameters.

Leaves were selected after tasseling, such that the leaf’s absolute position from the tassel could be tracked. Five to 6 leaves were measured from each plant, starting with the leaf closest to the tassel (leaf 1) and typically ending at the first ear leaf (e.g., leaf 5 or 6). The final model used represents an isotropic BRDF, where the output does not change when the object is rotated around the azimuth. So, what ultimately matters is the azimuthal difference between the incident and view vectors. See Fig. 1A to review the BRDF angular conventions used for the model. The mmBRDF was then quantified using our MFB sensor [17]. Data measured with the MFB sensor was fitted to a Mueller matrix model using the SCATMECH library [36]. To properly represent the BRDF of the leaves, a combination of 2 models were used: (a) a shadowed facet BRDF model, which described the interaction of light at the air–surface boundary of the leaf (specular reflection); and (b) a Lambertian BRDF model, which described the interaction of light with the leaf tissue (diffuse scattering).

The first model, a microfacet surface (specular) scattering model, was used to simulate the leaf’s epidermis as a function of incident angle (*θ_i_*), viewing angle (*θ_r_*), and azimuthal difference between the view and incident vectors (Δ*ϕ* = *ϕ_r_* − *ϕ_i_*) referred to as *f_f_* (*θ_r_*, *θ_i_*, Δ*ϕ*). Microfacets are perfect Fresnel reflecting elements that are small compared to the imaging system’s resolution. The distribution of reflected polarized light across the BRDF is proportional to distribution of the microfacets’ surface normal orientations. Typically, the most microfacets are oriented such that their surface normals align with the overall surface normal. Second, a volumetric Lambertian (diffuse) scattering model was used to represent the leaf’s tissue (e.g., the mesophyll) and is referred to as *f_d_* (*θ_r_*, *θ_i_*). The overall form of the model is given by

$$f\left(\theta_{r},\theta_{i},\Delta \phi \right)=af_{d}\left(\theta_{r},\theta_{i}\right)+bf_{f}\left(\theta_{r},\theta_{i},\Delta \phi \right)$$ (7)

where *a* and *b* are scale factors for the superposed model [17].

The mmBRDF model-fitting with SCATMECH outputs a few important parameters which were used to simulate data from field trials. These parameters are the scale factors *a* and *b*, dielectric constants *n* and *k*, the standard deviation of the surface slope used in a Smith shadowing function *w*, the standard deviation of the microfacet orientation angle assuming a Gaussian (normal) distribution *σ_RMS_*, and target reflectance *r*. By default, *a* = *b* = 1 and were not used as dynamic degrees of freedom when fitting the models. Rather, the epidermis’s reflectivity depends on *w*, *n*, and *k*, while *r* changed the reflectivity of the underlying Lambertian model.

### Correction model and Monte Carlo simulations

A shallow NN was trained with simulated data to accept any 2-band metric, calculated using the conventional *S*_0_ components, along with *S*_1_ and *S*_2_ of the lower reflectivity band for polarization correction. A Monte Carlo simulation was used to simulate *N* = 100,000 different illumination-, leaf-, and view-angle conditions, each with a range of parameter values with probability density functions (PDF) based on the MFB sensor and fitted SCATMECH model parameters.

Each variable in the simulation was assigned a PDF based on a uniform random distribution, defined as

$$f\left(x\right)=\left\{\;,\begin{array}{cc} \frac{1}{\beta -\alpha } & \text{if}\;\alpha \leq x\leq \beta ,\\ 0 & \text{otherwise}, \end{array}\right.$$ (8)

where the values of the PDF exist on the interval [*α*, *β*] for random variable *x*. Meanwhile, other parameters were simulated using a Gaussian PDF, defined as

$$f\left(x\right)=\frac{1}{\sigma \sqrt{2\pi }}\text{exp}\left[-\frac{{\left(x-\mu \right)}^{2}}{2\sigma^{2}}\right]$$ (9)

where *μ* is the mean and *σ* is the standard deviation.

Figure 5 below presents a workflow chart of the data pipeline and order of operations for merging the field trial data with the mmBRDF model data. The reflected Mueller matrix was simulated at 2 spectral bands with different reflectivity *r*. Due to the spectral invariance associated with most polarization phenomena—especially related to Fresnel reflections—*r* was the only parameter that depended on wavelength [11].

The PD functions and variables, for each parameter in the simulation, were as follows:

1. The illumination and view altitude angles, *θ_i_* and *θ_r_*, respectively, were randomized using the uniform PDF per Eq. 8 with *α* = 0^∘^ and *β* = 80^∘^.

2. The view angle’s azimuth, *ϕ_r_*, was simulated using the uniform PDF with *α* = 0^∘^ and *β* = 360^∘^.

3. The Lambertian reflectance *r*, specified for wavelengths *λ*_1_ and *λ*_2_, was denoted as *r_low_* and *r_high_*, respectively. Where *low* and *high* represent the low reflectivity and high reflectivity bands of the VI used. [label=)]

(a) Reflectance *r_low_* was randomly selected using a uniform PDF with *α* = 0.025 and *β* = 0.95.

(b) A random value *γ* for an arbitrary 2-band metric (e.g., RERR and GNDVI), defined as

$$\gamma =\frac{r_{\text{high}}-r_{\mathrm{low}}}{r_{\mathrm{low}}+r_{\text{high}}},$$ (10)

was selected from a uniform PDF with *α* = 0 and *β* = 1. The value of *r*_*high*_ was then calculated assuming *γ* positive *r*_*high*_ > *r*_*low*_ by

$$r_{\text{high}}=r_{\mathrm{low}}\frac{\left(1+\gamma \right)}{\left(1-\gamma \right)}.$$ (11)

4. Other optical and surface parameters were based on our assessment of materials using the MFB sensor’s measurements of live leaves, based on Table 1, as follows: [label=)]

**Table 1.** Mean and standard deviation of output parameters from SCATMECH model-fitting for 2 different B73 maize plants. These parameters were used to constrain values in Monte Carlo simulations.

	*n*	*k*	*σ*	*w*	*r*
Plant 1 mean	1.5298	-0.0169	45.5460	0.0575	0.1291
Plant 1 SD	0.0591	0.0388	1.6519	0.0224	0.0378
Plant 2 mean	1.4738	-0.0444	45.2617	0.0784	0.1139
Plant 2 SD	0.0547	0.0051	3.5753	0.0013	0.1388

(a) The value of *n* was selected from a normal PDF per Eq. 9 with parameters based on those identified from our mmBRDF measurements with a mean value of *μ* = 1.48 and *σ* = 0.1.

(b) Also, based on our mmBRDF measurements, the absorption of the first surface is low (*k* is small), a uniform PDF was used to model *k* with *α* =  −0.2 and *β* =  −0.01.(c) The microfacet angle standard deviation $\sigma_{\mathrm{RMS}}$ was modeled using a normal distribution with a mean value μ=45 degrees and standard deviation σ=2 degrees.

(d) The shadowing function standard deviation parameter *w* was modeled with a uniform PDF with *α* = 0.02 and *β* = 0.10.

The simulation returned the 4 × 4 mmBRDF at each randomly selected incidence-, view-, and leaf-parameter condition that the HIP system may observe [11,17,36,38]. These simulated data were used to quantify several network architectures to determine an optimal solution. Using MATLAB’s regression learner, we investigated exponential Gaussian regression models, linear regression models, decision trees, and shallow NNs using a variety of different optical channels (e.g., *S*_0_, *S*_1_, and *S*_2_ in the blue, green, red, and near-infrared), as well as the camera altitude, camera azimuth, sun altitude, and sun azimuth angles. The shallow NN was chosen as the final model for its simplicity, minimal channel requirements, and high accuracy.

### Field trial error analysis

**Fig. 6.**
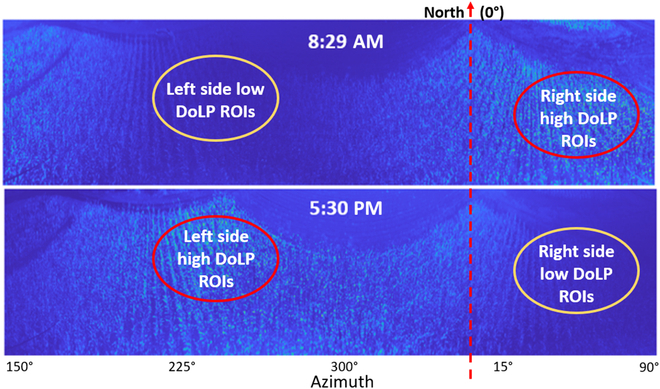
ROI selection using DoLP images versus time of day. HD ROIs selected from *t*_1_ = 8 : 29 AM (top) and *t*_2_ = 5 : 30 PM (bottom) DoLP images. The ROIs were selected from HD regions of the image (red circles) and the DoLP ROIs were obtained by extracting HD ROIs from a LD time of day (orange circles).

Regions of interest (ROIs) were extracted from 2 times of day, *t*_1_ and *t*_2_, in which each ROI experienced both a high DoLP (HD) and low DoLP (LD), respectively. This is depicted in Fig. 6 in which e.g., the right-hand side of the field contained a HD in the morning (*t*_1_ = 8:29 AM) and a LD in the late afternoon (*t*_2_ = 5:30 PM). As part of our analysis, the LD data was used as a form of comparative ground truth in that it generally corresponded to a low specular reflectance, as it was representative of both the mean and variance we observed in our transmission ground truth observations. The ROIs were selected exclusively on the HD side of both samples *t*_1_ and *t*_2_. To extract LD data, the HD ROIs were used in the opposing image; i.e., HD ROIs from the morning are extracted from evening data to acquire the LD ROI data. This allows us to do a one-to-one comparison for error calculation on the left-side and right-side FOV.

To enable a more direct comparison of ROIs before and after correction, the mean absolute error was calculated between the HD and LD ROIs by

$$\epsilon =\frac{1}{J}\sum_{j=1}^{J=10}‍{\left(\mu_{\mathrm{LD},j}-\mu_{\mathrm{HD},j}\right)}^{2},$$ (12)

where *J* is the total number of ROIs being evaluated and

$$\begin{array}{c} \mu_{\mathrm{LD},j}=\frac{1}{\mathrm{NM}}\sum_{m=1}^{M=5}‍\sum_{n=1}^{N=5}‍\gamma_{\mathrm{LD},j}\left(x_{n},y_{m}\right),\text{and} \end{array}$$ (13)

$$\begin{array}{c} \mu_{\mathrm{HD},j}=\frac{1}{\mathrm{NM}}\sum_{m=1}^{M=5}‍\sum_{n=1}^{N=5}‍\gamma_{\mathrm{HD},j}\left(x_{n},y_{m}\right), \end{array}$$ (14)

such that *x_n_* and *y_m_* are the ROI’s centroid coordinates, *γ*_*HD*,*j*_ is the *j*^th^ high DoLP 2-band ratio ROI, and *γ*_*LD*,*j*_ is the *j*^th^ low DoLP 2-band ratio ROI.

An additional metric that quantified the reduction in variance, experienced across all *J* = 10 ROIs, was calculated as

$$\epsilon_{\sigma }=\frac{1}{J}\sum_{j=1}^{J=10}‍\left(\frac{\sigma_{\mathrm{LD},j}}{\sigma_{\mathrm{HD},j}}\right),$$ (15)

where

$$\begin{array}{c} \sigma_{\mathrm{HD},j}=\sqrt{\frac{1}{\mathrm{NM}}\sum_{m=1}^{M=5}‍\sum_{n=1}^{N=5}‍{\left(\gamma_{\mathrm{HD},j}\left(x_{n},y_{m}\right)-\mu_{\mathrm{HD},j}\right)}^{2}},\text{and} \end{array}$$ (16)

$$\begin{array}{c} \sigma_{\mathrm{LD},j}=\sqrt{\frac{1}{\mathrm{NM}}\sum_{m=1}^{M=5}‍\sum_{n=1}^{N=5}‍{\left(\gamma_{\mathrm{LD},j}\left(x_{n},y_{m}\right)-\mu_{\mathrm{LD},j}\right)}^{2}}, \end{array}$$ (17)

where *σ*_*HD*,*j*_ and *σ*_*LD*,*j*_ are the standard deviations of the 2 band metric’s absolute error in *γ* for each pixel in the ROI. Generally, a reduction in both *ϵ* and *ϵ_σ_* should be observed when data are corrected versus uncorrected.

## Results

### pBRDF measurements and SCATMECH fitting

The SCATMECH model fitted parameter statistics from 2 different B73 maize plants are presented in Table 1 below. These parameters were measured and fitted using a *λ* of 550 nm. The 2 models generally agree with each other and substantiates our ability to create a generalized library of mmBRDF models for maize leaves.

### Correction model simulations

**Fig. 7.**
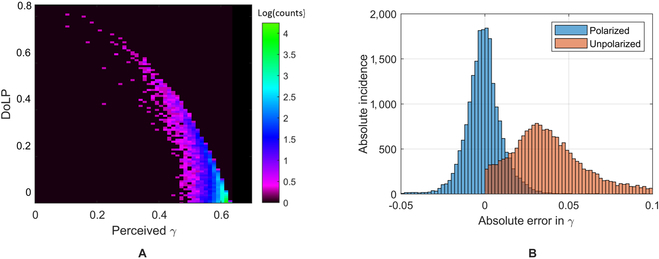
Simulation histograms. Histogram of simulation outputs for (A) DoLP versus the sensor’s perceived *γ*, as calculated from *S*_0, *low*_ and *S*_0, *high*_ (e.g., without correction) for a true *γ* = 0.62. presented with the color axis in a log-scale and units of measurement as log(counts); and (B) the absolute error across the range of the Monte Carlo simulation parameters using a model that accepts only unpolarized components (orange) versus polarized components (blue).

The final model accepted *γ*, as calculated conventionally using the uncorrected *S*_0_ reflectivities from (*r_high_*) and (*r_low_*) reflecting wavelengths per Eq. 10, as well as the low reflectivity *S*_1_ (*S*_1,*low*_) and *S*_2_ (*S*_2,*low*_) Stokes parameters. Figure 7A depicts the results of the simulated DoLP versus the sensor’s perceived (uncorrected) value of *γ*, assuming that the plant has an constant *γ* = 0.62. We can see that as the degree of polarization increases, the value of *γ* decreases. Furthermore, there is a region where high error and low polarization exists such that the DoLP is close to 0 with a range of *γ* from approximately 0.45 to 0.6 and is the result of retroreflective hot spots where *μ_i_* · *μ_r_* ≈ 0. It should be noted that our technique aims at correcting pixels with high DoLP and erroneous *γ* but that items positioned in retroreflection (e.g., reflectivity hot spots [46]) will require further development to correct. This behavior is also observed in Fig. 7B, which shows a histogram of the absolute incidence versus the absolute error in *γ* for 2 algorithms: (a) using our optimal shallow NN with polarization mean error of 1.9E-4 and standard deviation of 0.019; and (b) a shallow NN, trained using only the sensor’s perceived *S*_0_ components with mean error of 0.049 and standard deviation of 0.050. Generally, the uncorrected data’s absolute error is positive because specularly reflected light will always decrease the perceived value of *γ* due to the increased radiance at the low-reflectivity spectral band, relative to the high-reflectivity spectral band.

### Spectral ground truth

**Fig. 8.**
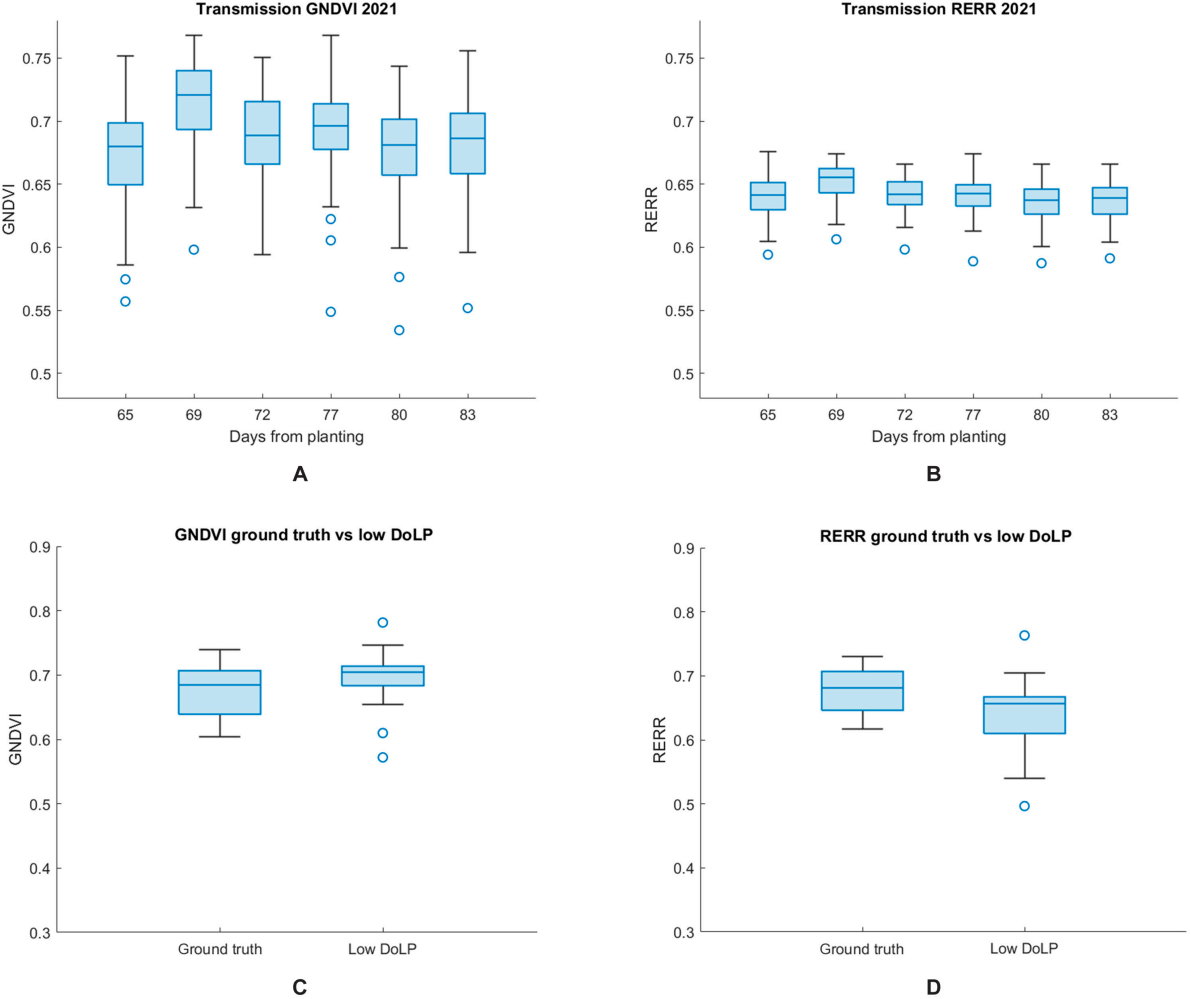
Ground truth box charts with low DoLP comparison. (A and B) Box charts representing the ground truth GNDVI and RERR, respectively, with the x-axis presented in Julian days since planting. (C and D) Box charts comparing the data distributions of ground truth data and low DoLP region data for GNDVI and RERR, respectively.

Ground truth measurements, captured with the handheld device, are provided in Fig. 8A and B below for GNDVI and RERR, respectively, taken across the field (all 80 plots, or 400 leaf transmission measurements per day), for each day. Generally, plants behaved uniformly across the field and across time, which supports a key assumption to our study that underlying variation, measured by the HIP sensor, will be caused by illumination and geometrical effects, as opposed to variations in the canopy caused by nutrient deficiencies, herbicide overspray, or diseases.

Evaluation of the correction network was achieved by comparing corrected and uncorrected ROIs from low-DoLP and high-DoLP regions. In effect, we made the assumption that the low-DoLP regions can serve as a proxy for ground truth targets because these regions have low specular reflectance. Figure 8C and D demonstrates the reliability of this assumption by providing a comparison between the mean and variance of GNDVI and RERR calculated from ground truth data and low DoLP ROI data.

### Correction model validation using HIP field trial data

**Fig. 9.**
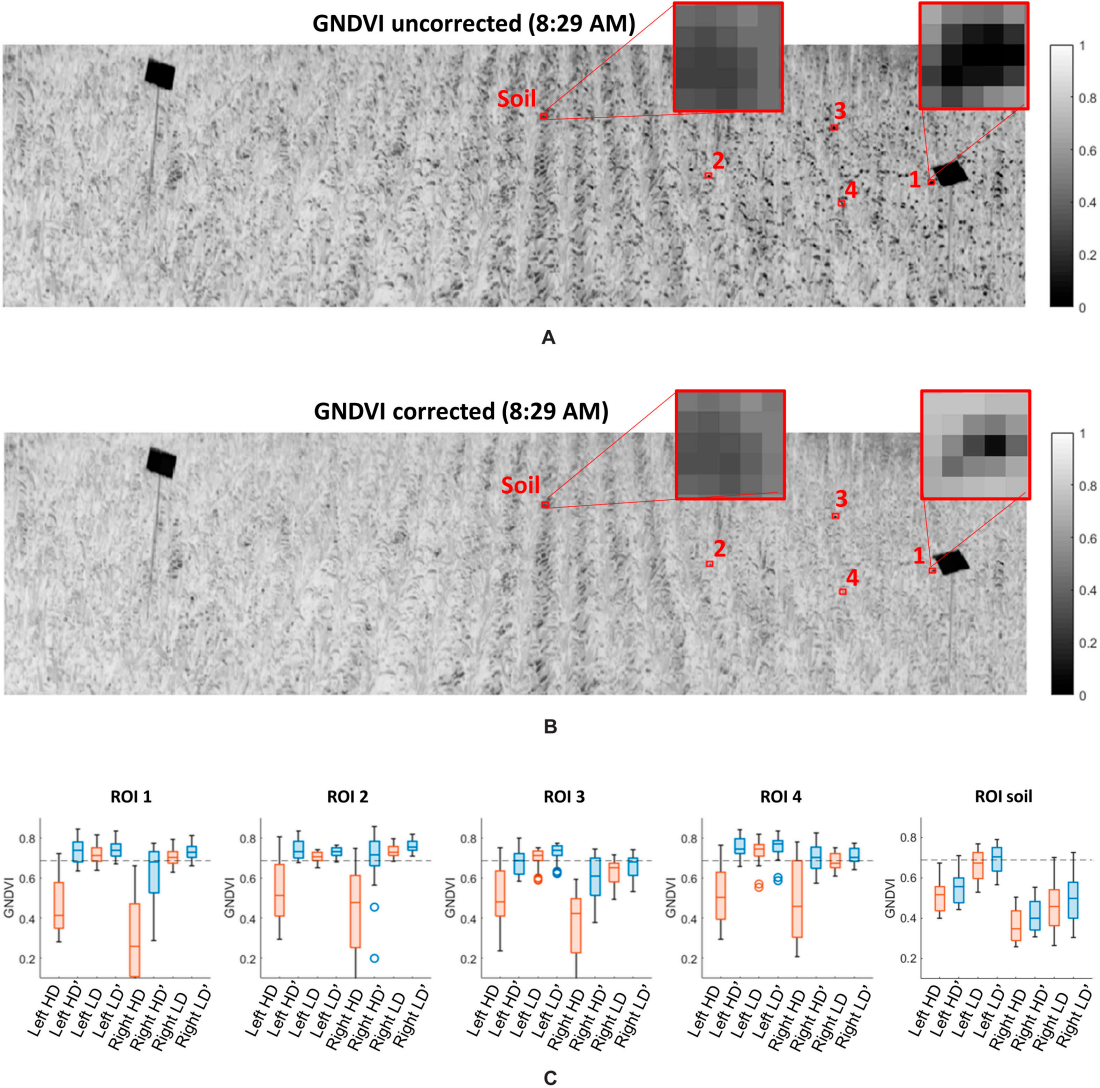
Comparison of GNDVI grayscale images, captured at 8:29 am, before and after correction, focusing on an area of the field with high DoLP. (A) Uncorrected GNDVI image. Note the black spots, these are primarily the result of glare. (B) The same GNDVI image after polarization correction. Note the field looks more uniform and many of the black spots have faded or disappeared entirely with the exception of ROI soil. (C) Box charts comparing small ROIs from areas with high DoLP (HD) and low DoLP (LD) before and after correction. The red boxes are before correction, blue boxes are after correction, and the black dotted line displays the mean GNDVI from ground truth data.

**Fig. 10.**
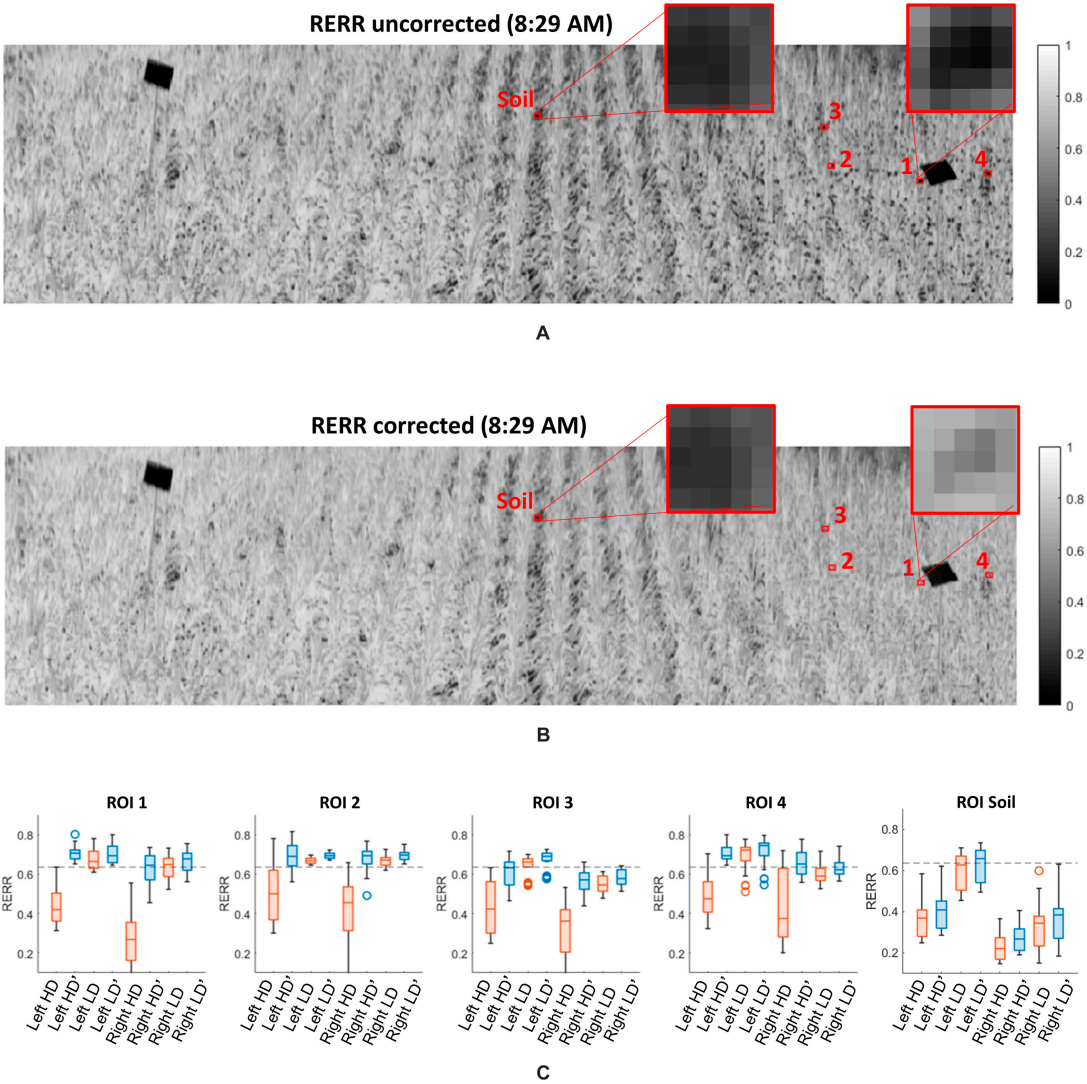
Comparison of RERR grayscale images, captured at 8:29 am, before and after correction, focusing on area of the field with high DoLP. (A) Uncorrected RERR image. Note the black spots, these are primarily the result of glare. (B) The same RERR image displayed after correction. Note the field looks more uniform and many of the black spots have faded or disappeared entirely with the exception of ROI soil. (C) Box charts comparing small ROIs from areas with high DoLP (HD) and low DoLP (LD) before and after correction. The red boxes are before correction, blue boxes are after correction, and the black dotted line displays the mean RERR from ground truth data.

For our field trial geometry, location, and sensor viewing angles, image samples captured in the morning and afternoon had higher DoLP than those measured at noon, when the sun was highest in the sky (closest to zenith) (see Fig. 1B). A single data cube from approximately 8:30 AM was used to calculate GNDVI and RERR images, as per Figs. 9A and 10A, respectively. These VI images were calculated using only *S*_0_ (unpolarized) data without correction.

As described previously, the correction network accepts any 2-band metric, *γ*, along with the *S*_1_ and *S*_2_ data of the VI (*γ*) band with the lower *S*_0_ reflectivity. For GNDVI, the *S*_0_ components corresponding to *λ*_1_ = 530 ± 10 nm and *λ*_2_ = 780 ± 10 nm were used to calculate *γ_GNDVI_*, along with *S*_1_ and *S*_2_ of the low reflectivity (*λ*_1_) spectral band. For RERR, the *S*_0_ components corresponding to *λ*_1_ = 700 ± 10 nm and *λ*_2_ = 780 ± 10 nm, along with the *S*_1_ and *S*_2_ parameters of *λ*_1_. The corrected VI images for GNDVI and RERR are presented in Figs. 9B and 10B, respectively. Additionally, box charts comparing 5 different 5 × 5 ROIs, from the HD and LD areas of the GNDVI and RERR images, are presented in Figs. 9C and 10C. The first 4 ROIs were selected from high-DoLP areas, and the fifth, ROI soil, was included to demonstrate that the correction network is minimally influenced by background (nonleaf) pixels. Finally, the black dotted lines represent our target VI, calculated using data acquired with our ground truth device.

Using these ROIs, the error *ϵ* from Eq. 12 and *ϵ_σ_* from Eq. 15 are summarized in Table 2 for GNDVI and RERR metrics. Overall, the MSE was reduced by a factor of 12 to 80, while the standard deviation was reduced by a factor of 1.5 to 2.7.

**Table 2.** Error summary before and after correction, as calculated for the left and right sides of the FOV. This excludes the soil pixel ROIs presented in the last box charts of Figs. 9C and 10C.

Vegetation index	Left side	Right side
GNDVI uncorrected (*ϵ*)	3.24%	9.06%
GNDVI corrected (*ϵ*)	0.13%	0.60%
GNDVI uncorrected (*ϵ_σ_*)	3.94	5.65
GNDVI corrected (*ϵ_σ_*)	1.45	3.73
RERR uncorrected (*ϵ*)	2.80%	6.56%
RERR corrected (*ϵ*)	0.23%	0.08%
RERR uncorrected (*ϵ_σ_*)	3.38	4.57
RERR corrected (*ϵ_σ_*)	1.46	2.05

## Discussion

The results from ground truth data in Fig. 8A and B provide validation for our assumption that the VIs used were both spatially and temporally uniform. For GNDVI and RERR, the standard deviation of the daily average was 0.016 and 0.013, respectively, and the standard deviation across the field was calculated to be 0.028 and 0.026 for GNDVI and RERR, respectively. Furthermore, we made an assumption that areas of the field with low DoLP in the morning can serve as a secondary means of ground truth for those same areas showing high DoLP in the evening, allowing us to provide a pixel-to-pixel comparison of ROIs before and after correction was applied. Figure 8C and D validates this assumption by presenting box charts of ground truth data and ROI data taken from low DoLP regions of the field. For GNDVI, the mean of both ground truth and low DoLP data were 0.689 and 0.669, respectively, and for RERR the mean ground truth and low DoLP values were 0.636 and 0.621, respectively.

Results indicated that using polarimetry is a viable solution to reduce the confounding impact caused by specularly reflected light. Notable improvements were observed between the uncorrected and corrected VI imagery in Fig. 9A and B for GNDVI, respectively, or Fig. 10A and B for RERR, respectively. As expected, specularly reflected light in the high-DoLP areas of an uncorrected VI image caused a reduction in both VIs, as noted by dark spots in the 2-band imagery. These dark spots are primarily the result of glare coming off the leaves, which can be modeled by including both the specular and diffuse scattering components of Eq. 10 such that

$$\gamma =\frac{\left(r_{\text{high},L}+r_{\text{high},S}\right)-\left(r_{\mathrm{low},L}+r_{\mathrm{low},S}\right)}{\left(r_{\mathrm{low},L}+r_{\mathrm{low},S}\right)+\left(r_{\text{high},L}+r_{\text{high},S}\right)},$$ (18)

where the subscript L and S represent the Lambertian and specular components of the reflectivity, respectively, at a given scattering angle within the mmBRDF function. Assuming we have (a) a Lambertian leaf volume that is highly absorptive and reflective at *λ*_1_ and *λ*_2_, such that *r*_*low*,*L*_ ≈ 0 and *r*_*high*,*L*_ >> *r*_*low*,*L*_), providing a high VI value that is independent of scattering angle; and (b) identical dielectric constants at *λ*_1_ and *λ*_2_, such that at a given scattering angle *r*_*high*,*S*_ ≈ *r*_*low*,*S*_ and *r*_*high*,*S*_ and *r*_*low*,*S*_ increase toward the specular direction. Under these assumptions, any increase in the specular reflectivity, caused by an increasing scattering angle, will decrease *γ* since the denominator will increase while the numerator remains unchanged. This behavior is also supported by our simulation results per Fig. 7A, in that increasing DoLP, corresponding to higher specular reflections, reduced the sensor’s measured (perceived) value of *γ*.

The improvement for RERR and GNDVI were similar, despite using the same correction model. We can see that the variance of the ROI data after correction is slightly greater than ground truth data; however, it still falls within reasonable margins for most remote phenotyping scenarios. Referring to Table 2, we see an improvement of an order of magnitude or more in the mean error *ϵ* for both VIs, and a reduction spanning 1.5 to 2.7 in their standard deviation *ϵ_σ_*. This is similar to the standard deviation’s reduction observed in the simulations per Fig. 7B, which decreased by a factor of 2.6 after correction. A reduction of error and variance was also observed in the ROI’s box plots per Figs. 9C and 10C. This further confirms the spectral invariance hypothesis and may imply that the same correction network can work for any 2-band metric.

Finally, the ROI containing soil pixels was included in Figs. 9C and 10C to demonstrate that the correction has minimal effect on background pixels with nonglare-induced reductions in their VI values. Specifically, the mean VI before and after correction were measured to be 0.49 and 0.53 for GNDVI, respectively, and 0.35 and 0.37 for RERR, respectively. Thus, the correction method increased the value by approximately 8.7% for GNDVI and 11.9% for RERR, but since the DoLP of these pixels is low, it demonstrates a potential advantage that the correction method will not attempt to overcorrect the data based simply on a low VI value.

Maize leaves were measured with our in-lab MFB mmBRDF sensor. Results were used to fit modeling parameters for SCATMECH with mean dielectric constants *n* = 1.495 and *k* =  −0.031, surface roughness *σ* = 45.40 microns, shadowing deviation *w* = 0.068, and Lambertian reflectance *r* = 0.1215 at 550 nm. These parameters informed a Monte Carlo simulation, also performed using SCATMECH, the output of which was used to train a correction model. The correction model was based on a shallow NN that accepted the measured (perceived) value of *γ*, as calculated by the uncorrected *S*_0_ at *λ*_1_ and *λ*_2_, as well as the *S*_1_ and *S*_2_ Stokes parameters at the low reflectivity wavelength (*λ*_1_). Validation was performed through spectropolarimetric image data of maize captured in the field using a mast-mounted HIP. The data were then curated and sent into the correction network in the form of a 2-band metric (RERR, GNDVI, etc.). The performance of the correction network was demonstrated in 2 ways. One visually, by calculating a VI across the FOV and presenting the VI image before and after correction. The other statistically, by extracting 5 ROIs from high and 5 from low DoLP regions, from 2 different times of day. ROIs were then compared before and after polarization correction.

The correction method demonstrated promising results that support our use of polarimetry to reduce the impact of glare, for phenotypic imaging, in uncontrolled environments. The correction method was shown to reduce the average absolute GNDVI and RERR error by a factor of 33 and the average standard deviation by a factor of 2.1. These results support polarimetry playing a critical role in the future success of multi- and hyperspectral sensor systems for high-throughput phenotyping applications in the field.

Hypothetically, once we have an established library of mmBRDF leaf and correction models, a user would only need to obtain the HIP data for correction to take place. The current algorithm is relatively simple, so it is unlikely to be a bottleneck in high-throughput phenotyping applications. The model presented here was trained on B73 maize. In future work, we intend train new NNs with different maize varieties to explore whether or not this correction network can be applied across different genotypes. Furthermore, the data presented here were selected from clear, sunny days. Assessing the impacts of clouds, and other weather conditions, on the correction network will also be the focus of future work, as well as generally correcting the hyperspectral reflectance. Our long-term goal is to publish a library of mmBRDF leaf and correction models for different genotypes of maize and eventually other crops like soybean and cucurbits.

## Data Availability

Data underlying the results presented in this paper are not publicly available at this time but may be obtained from the authors upon reasonable request.
